# Establishing a Novel *E. coli* Heterologous Secretion Expression System Mediated by mScarlet3 for the Expression of a Novel Lipolytic Enzyme

**DOI:** 10.3390/biom15060842

**Published:** 2025-06-09

**Authors:** Jun Yang, Mingjun Yang, Huichen Liu, Xinyu Liu, Fei Wang, Wenqiang Li, Yang Liu, Chao Zhai, Lixin Ma

**Affiliations:** State Key Laboratory of Biocatalysis and Enzyme Engineering, Hubei Key Laboratory of Industrial Biotechnology, School of Life Sciences, Hubei University, Wuhan 430062, China; youcojun@stu.hubu.edu.cn (J.Y.); 202411107010122@stu.hubu.edu.cn (M.Y.); liuhc0223@stu.hubu.edu.cn (H.L.); 202221107011768@stu.hubu.edu.cn (X.L.); wangfei@hubu.edu.cn (F.W.); li-wenqiang@hubu.edu.cn (W.L.); lyang@hubu.edu.cn (Y.L.)

**Keywords:** lipolytic enzyme, mScarlet3, *E. coli* secretion expression

## Abstract

Our previous study demonstrated that an *Escherichia coli* heterologous secretion expression system, mediated by superfolder green fluorescent protein (sfGFP) mutants, significantly enhances recombinant lipase yield and reduces large-scale production costs. In this study, we identified mScarlet3, a fast-folding fluorescent protein, as another effective mediator of secretion expression in *E. coli*. A novel lipolytic enzyme, named LipHu6, was identified through sequence alignment. Secretion expression of LipHu6 was achieved by fusing mScarlet3 to either its N- or C-terminus. The specific activity of mScarlet3-LipHu6 reached 669,151.75 U/mmol, slightly surpassing that of LipHu6 alone (646,682.69 U/mmol) and markedly exceeding that of sfGFP_(-15)_-LipHu6 (492,432.39 U/mmol). Notably, N-terminal mScarlet3 fusion had no impact on LipHu6 hydrolytic activity toward short-chain *p*-nitrophenyl fatty acyl esters (C2–C8). In contrast, mScarlet3-LipHu6 exhibited approximately 1.5- and 1.7-fold increases in hydrolytic activity toward *p*-nitrophenyl palmitate (p-NPP, C16) and *p*-nitrophenyl stearate (p-NPS, C18), respectively. In conclusion, this study establishes a novel *E. coli* heterologous secretion expression system mediated by mScarlet3, offering a highly efficient and cost-effective strategy for the large-scale production of lipolytic enzymes.

## 1. Introduction

Lipolytic enzymes (EC 3.1.1.–) encompass a diverse group of α/β hydrolases that catalyze the hydrolysis or formation of ester bonds [1]. These enzymes are classified into two main categories, esterases (EC 3.1.1.1) and lipases (EC 3.1.1.3), which differ in their kinetic properties and substrate chain length preferences [2]. Esterases catalyze the hydrolysis of small, partially water-soluble ester-containing molecules, such as short-chain acylglycerols. In contrast, lipases exhibit optimal activity toward long-chain triglycerides, which are insoluble in aqueous environments and display distinctive interfacial activation [3,4,5]. Lipolytic enzymes are widely used in asymmetric synthesis of pharmaceutical and fine chemical industry [6,7], biodiesel production [8,9], waste treatment [10], food industry [11,12], etc. Considering the extensive industrial applications of lipolytic enzymes, developing a versatile heterologous expression platform is critical for their large-scale production at low cost and high yield. Secretion expression is a commonly used strategy in industry with advantages such as high product titer and low cytotoxicity, as well as simplified downstream purification process [13]. Numerous successful cases of heterologous secretion expression were achieved in Gram-positive strains such as *Bacillus sutilis*, and eukaryotic hosts, such as *Pichia pastoris*, *Saccharomyces cerevisiae*, *Aspergillus oryzae*, Chinese hamster ovary cells, HEK 293 cells, etc. [14]. Through genetic manipulations of the host cells and optimization of secretion tags, more and more recombinant proteins were expressed extracellularly with Gram-negative strains such as *E. coli* in recent years [15,16]. Previously, we reported high-level secretion expression of *Rhizomucor miehei* (RM) lipase using an *E. coli* secretion system mediated by a sfGFP mutant with 15 net negative charges, (sfGFP_(-15)_) [17]. RM lipase was efficiently secreted with sfGFP_(-15)_ fused to either its N- or C-terminus, suggesting that sfGFP_(-15)_ mediates secretion through a mechanism distinct from canonical signal peptides. We proposed that the β-barrel structure and net negative charges distributed on the molecular surface are critical for this system. Furthermore, the lipolytic activity of RM lipase may disturb the cellular phospholipid bilayer, facilitating the release of the target protein from the cytoplasm to the extracellular environment. However, the precise mechanism remains unknown. Numerous proteins in nature possess a β-barrel structure, including transmembrane β-barrel proteins [18] and red fluorescent protein (RFP) homologs from corals or other Anthozoa species [19,20]. Among them, mScarlet showed unique features. It is a cysteine-free monomeric red fluorescent protein with fast and complete folding efficiency. This red fluorescent protein has not evolved from a natural ancestor. On the contrary, it is a synthetic gene designed with mCherry and multiple other naturally occurring RFPs and chromo proteins as templates. Therefore, it overcame the shortcoming of RFPs that tend to form dimmer and remained monomer after maturation. After multiple site-directed mutageneses and several rounds of random mutagenesis with this synthetic template, mScarlet and two variants with bright and fast-folding property were obtained [21]. Subsequently, Gadella et al. obtained mScarlet3 based on the evolution of mScarlet in 2023 [22]. Targeted mutations were introduced within the β-barrel structure near threonine-74, significantly enhancing the brightness and folding rate of mScarlet3.

To investigate the role of the β-barrel structure in the secretion expression of lipolytic enzymes using *E. coli* as the host, we utilized mScarlet3 as a fusion tag to mediate the secretion expression of the lipolytic enzyme LipHu6. The result indicated mScarlet3 successfully mediated the secretion expression of LipHu6 and outperformed sfGFP_(-15)_. Notably, the fusion protein with mScarlet3 at either the N- or C-terminus was effectively expressed, whereas sfGFP_(-15)_ was functional only when fused to the N-terminus of LipHu6. Furthermore, mScarlet3 had a lesser impact on the enzymatic activity of LipHu6 compared to sfGFP_(-15)_. Collectively, this study introduces a novel fluorescent protein for the highly efficient secretion expression of lipases.

## 2. Materials and Methods

### 2.1. Bacteria, Plasmids, Media and Reagents

*E. coli* DH5α for gene cloning was stored in our lab. *E. coli* BL21 (DE3) for protein expression was purchased from TransGen Biotechnology (Beijing, China). Plasmids pET23a, pET28a and pET23a-sfGFP_(-15)_ was stored in our lab. Luria–Bertani (LB) were prepared as described in the Manual of Molecular Cloning [23]. The substrates 4-nitrophenyl acetic acid (*p*-NPA), 4-nitrophenyl butyrate (*p*-NPB), 4-nitrophenyl octanoate (*p*-NPO), 4-nitrophenyl laurate (*p*-NPL), 4-nitrophenyl palmitate (*p*-NPP) and 4-nitrophenyl stearate (*p*-NPS) were purchased from Macklin Biochemical Co., Ltd. (Shanghai, China). All other chemicals were analytical reagents.

### 2.2. Expression and Recovery of LipHu6 Fused with sfGFP_(-15)_

The LipHu6 gene was originated from the IMG metagenomic database (IMG Submission ID Ga0070767_11070263). The ORF encoding LipHu6 was synthesized by Sangon (Shanghai, China) and cloned into pET28a and pET23a-sfGFP_(-15)_ vector through TLTC method [24] to generate expression vectors pET28a-LipHu6, pET23a-sfGFP_(-15)_-LipHu6 and pET23a-LipHu6-sfGFP_(-15)_. To facilitate purification, a 6× His-tag was fused to the C-terminus of the fusion proteins. Recombinant plasmids were verified by Sanger sequencing and transformed into *E. coli* BL21 (DE3). To induce target gene expression, transformants were cultured at 37 °C until reaching an OD_600_ of 0.6–0.8, then induced with 0.5 mM isopropyl-β-D-thiogalactopyranoside (IPTG) at 18 °C for 24 h. To recover the recombinant protein, cells were harvested by centrifugation at 5000× *g* for 10 min, resuspended in 1 mL of phosphate-buffered saline (PBS), and incubated at 4 °C overnight.

### 2.3. Expression and Recovering of LipHu6 Fused with mScarlet3

The ORF encoding mScarlet3 was synthesized by Sangon (Shanghai, China) and replaced the sfGFP_(-15)_ coding fragments in pET23a-sfGFP_(-15)_-LipHu6 and pET23a-LipHu6-sfGFP_(-15)_ vectors through TLTC method [24] to generate expression vectors pET23a-mScarlet3-LipHu6 and pET23a-LipHu6-mScarlet3, respectively. To facilitate the purification, a 6× His tag was fused to the C-termini of the fusion proteins. The recombinant plasmids were verified by Sanger sequencing, followed by transforming into *E. coli* BL21 (DE3). The procedure of the induction expression of the target genes was carried out as described in Section 2.2.

### 2.4. Purification of the Recombinant Proteins with Ni-Affinity Chromatography

Cells were collected and resuspended in lysis buffer (50 mM Tris-HCl; 200 mM NaCl; 50 mM NaH_2_PO_4_; 25 mM Imidazole; 10% Glycerol, pH 8.0). Samples were ultrasonicated to break the cells. The crude cell lysate was collected after centrifugation at 13,000 rpm for 10 min. The sample was then centrifugated at 13,000 rpm for 10 min, and the supernatant was applied to Ni-NTA beads for affinity purification. The column was washed twice with 2 column volume of wash buffer (50 mM Tris-HCl; 200 mM NaCl; 50 mM NaH_2_PO_4_; 10 mM Imidazole, pH 8.0). One column volume of elution buffer (50 mM Tris-HCl; 200 mM NaCl; 50 mM NaH_2_PO_4_; 50 mM Imidazole, pH 8.0) was used to recover the target protein. The sample was then collected and dialyzed with a Millipore 10 kDa cut-off membrane at 4 °C to remove ions and salts, followed by resuspending with storage buffer (50 mM Tris-HCl, pH 8).

### 2.5. Sodium Dodecyl Sulfate Polyacrylamide Gel Electrophoresis (SDS-PAGE)

Protein samples were separated via SDS-PAGE using 10~15% (*w*/*v*) polyacrylamide gel, followed by staining with Coomassie Brilliant Blue G250. The samples were separated via SDS-PAGE using 12% (*w*/*v*) polyacrylamide gels, followed by staining with Coomassie Brilliant Blue R-250. The protein concentrations were determined using the Bradford kit (Beyotime, Shanghai, China); bovine serum albumin was used as the standard.

### 2.6. Analysis of the Enzymatic Activity of LipHu6

To investigate the activity of the recombinant LipHu6 with or without mScarlet3 tag, *p*-NPA, *p*-NPB, *p*-NPO, *p*-NPL, *p*-NPP and *p*-NPS were used as the substrates. In a standard assay, the reaction mixture contained 40 μL of the substrate (0.5 mM), 940 μL of Tris-HCl buffer (50 mM, pH 8.0) and 20 μL of diluted enzyme solution. The mixture was incubated at 40 °C for 5 min and the reaction was terminated with 60 μL of 20% Trichloroacetic Acid. Subsequently, 300 μL of 0.5 M NaOH was added and the sample was centrifuged at 1000× *g* for 1 min, followed by measuring the absorbances at 405 nm. All experiments were conducted in triplicate and 4-Nitrophenol was used to plot the standard curve. One unit (U) of lipase activity was defined as the amount of enzyme that released 1 μmol of *p*-NP per min under the assay conditions.

### 2.7. Analysis of Enzymatic Characteristics

The optimum pH of LipHu6 with or without fluorescent protein tags was determined by measuring enzyme activity at 40 °C in the pH range from 3.0 to 11.0. The optimum temperatures of LipHu6 with or without fluorescent protein tags were determined in the range of 0 to 80 °C at pH 8.0.

### 2.8. Kinetic Analysis

To obtain kinetic parameters for LipHu6 with mScarlet3 fused to its N- or C-terminus, 0.25 to 2.5 mmol/L of *p*-NPB was used as the substrate and reacted at pH 8.0, 40 °C for 5 min. Initial rate data were fitted to a Michaelis–Menten equation, and the *K_M_* and *k_cat_* values were calculated from the slope and y-intercept of the double-reciprocal plot. All reactions were performed in triplicate.

## 3. Results

### 3.1. The Bioinformation Analysis of mScarlet3 Protein

The amino acid sequence of mScarlet3 protein was indicated in Supplementary Figure S1. It is a protein of approximately 25.8 kDa with pI of 6.01. It has 13 arginines, 18 lysines, 15 aspartates and 19 glutamates. The distribution of positive/negative charges and hydrophobic/hydrophilic residues on the surface of mScarlet3 and sfGFP_(-15)_ were calculated with UCSF ChimeraX version 1.7 (San Francisco, CA, USA) (Supplementary Figure S2). Both proteins demonstrated typical β-barrel structure and possessed strong hydrophilic surfaces. On the other hand, sfGFP_(-15)_ had obviously more negative charges at its surface than mScarlet3.

### 3.2. The Secretion Expression of mScarlet3 Protein with E. coli as the Host

**Figure 1:**
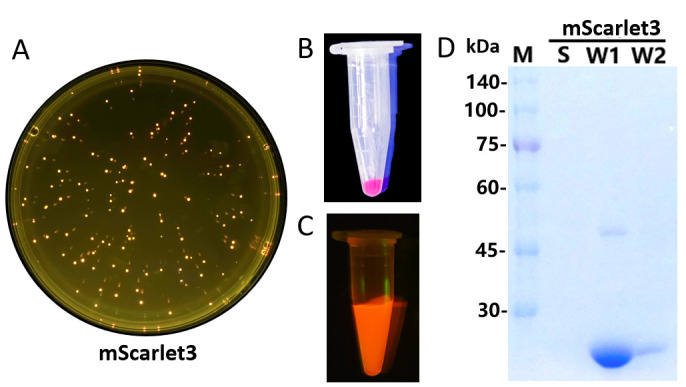
The secretion expression of mScarlet3 protein with *E. coli* as the host. (**A**) The colonies of *E. coli* BL21 (DE3) bearing pET23a-mScarlet3 on LB plates supplemented with ampicillin; (**B**) the cells of *E. coli* BL21 (DE3) bearing pET23a-mScarlet3 collected from shake-flask fermentation; (**C**) the supernatant of cells washed once with PBS. (**D**) SDS-PAGE analysis of mScarlet3 secreted by the recombinant strain. M denotes protein molecular weight marker (the size of each band was indicated on the left). S stands for the supernatant collected from shake-flask fermentation; W1 stands for the supernatant from cells washed with PBS once; and W2 stands for the supernatant from cells washed with PBS twice.

To evaluate the secretion expression of mScarlet3 in *E. coli*, the plasmid pET23a-mScarlet3 was transformed into *E. coli* BL21 (DE3) and plated on Luria–Bertani (LB) agar plates supplemented with ampicillin. Pink fluorescence was observed in the colonies under blue light after 16 h of incubation (Figure 1A), indicating leaky expression of the target protein. Subsequently, a single colony was selected for shake-flask fermentation. Following 16 h of induction with IPTG, no target protein was secreted directly into the cell culture, but the cells exhibited bright pink fluorescence (Figure 1B). After washing with phosphate-buffered saline (PBS) at 4 °C overnight, the fluorescence intensity of the cells decreased, while the washing buffer exhibited increased pink fluorescence (Figure 1C), suggesting that recombinant mScarlet3 was translocated into the buffer. SDS-PAGE analysis confirmed that most of the recombinant mScarlet3 was released from the cells after PBS washing once (Figure 1D). These results suggest that mScarlet3 can be secreted by the *E. coli* heterologous expression system.

### 3.3. The Secretion Expression of Lipase Hu6 Fused with Fluorescent Protein Tags

**Figure 2:**
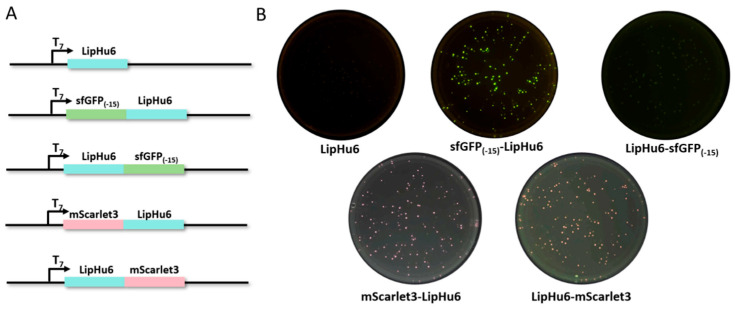
The structure of the proteins. (**A**) Schematic illustrating the structure of the fusion protein. The ORF of LipHu6 is labeled in blue. The ORF of sfGFP_(-15)_ is labeled in green while The ORF of mScarlet3 is pink. (**B**) The colonies of *E. coli* BL21 (DE3) bearing the expression vectors on LB plates supplemented with ampicillin.

Plasmids pET28a-LipHu6, pET23a-sfGFP_(-15)_-LipHu6, pET23a-LipHu6-sfGFP_(-15)_, pET23a-mScarlet3-LipHu6, and pET23a-LipHu6-mScarlet3 (Figure 2A) were transformed to *E. coli* BL21 (DE3). After approximately 16 h incubation at 37 °C, the colonies bearing pET23a-mScarlet3-LipHu6 and pET23a-LipHu6-mScarlet3 displayed obvious pink fluorescence under blue light in comparison with the negative control (colonies bearing pET28a-LipHu6) (Figure 2B). On the other hand, colonies harboring pET23a-sfGFP_(-15)_-LipHu6 displayed bright green fluorescence while colonies with pET23a-LipHu6-sfGFP_(-15)_ showed no fluorescence (Figure 2B).

**Figure 3:**
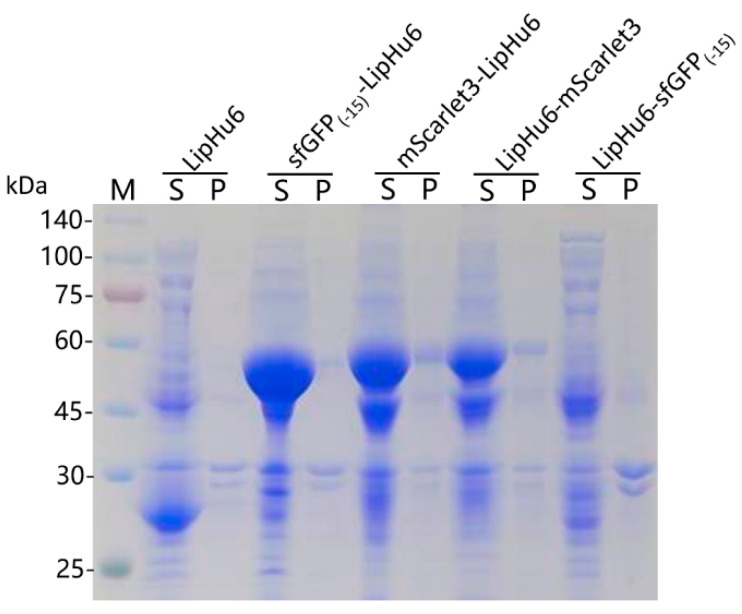
SDS-PAGE analysis of the recombinant proteins. The names of the target proteins are labeled on the top of the figure. M denotes the protein molecular weight marker (the size of each band was indicated on the left). S stands for the supernatant of the cell lysate; P stands for the pellet of the cell lysate.

A single colony was picked from each plate for shake-flask fermentation. Consistent with the result of plate assay, SDS-PAGE indicated that a band with the predicted molecular weight of the target protein was detected with *E. coli* strains bearing pET28a-LipHu6, pET23a-sfGFP_(-15)_-LipHu6, pET23a-mScarlet3-LipHu6, pET23a-LipHu6-mScarlet3. On the contrary, no obvious band representing LipHu6-sfGFP_(-15)_ was detected (Figure 3).

**Figure 4:**
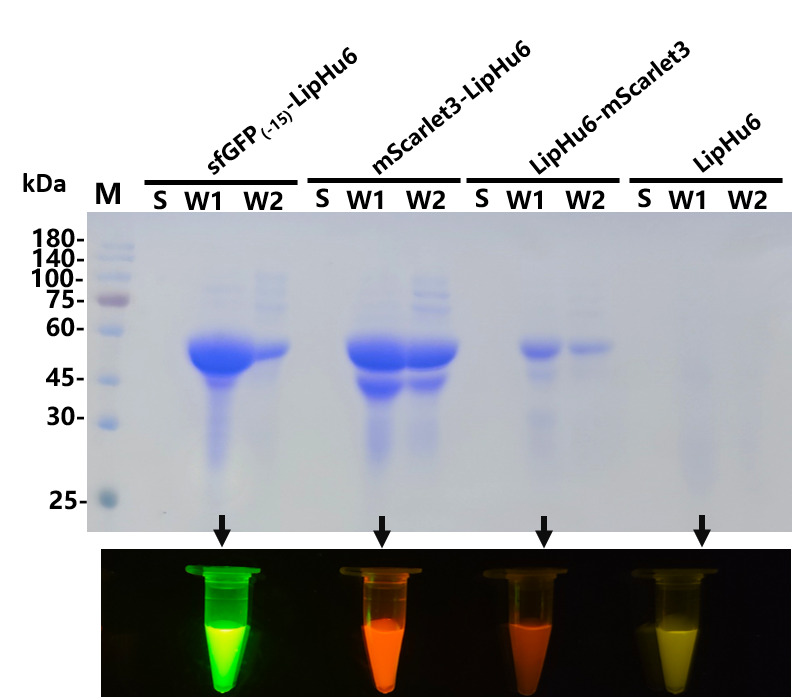
SDS-PAGE was performed to investigate the effect of fluorescent protein tags on the secretion expression of LipHu6. M denotes the protein molecular weight marker (the size of each band was indicated on the left). S stands for the supernatant shake-flask fermentation, W1 stands for the cells washed with PBS once and W2 stands for the cells washed with PBS twice.

No target protein was secreted to the cell culture directly (Figure 4). Therefore, the cells were collected and washed with PBS. Consistent with previous results, the green and pink fluorescence was transferred to the washing buffer after the incubation (Figure 4). The results of SDS-PAGE confirmed that sfGFP_(-15)_ and mScarlet3 mediated the secretion expression of LipHu6 successfully. A band with predicted size was detected in the supernatant after the cells were washed once. With mScarlet3-LipHu6, more target protein was obtained when the cells were washed again. On the other hand, no target protein was detected in the supernatant for the intracellular expression of LipHu6 after washing with PBS (Figure 4). The activity of the enzymes recovered by washing with PBS was evaluated. The result indicated the specific activity of mScarlet3-LipHu6 was 605,538.49 U/mmol, which was obviously higher than that of sfGFP_(-15)_-LipHu6 and LipHu6-mScarlet3, which was 497,860.72 U/mmol and 549,600.40 U/mmol, respectively.

### 3.4. The Effect of Fluorescent Protein Tags on the Activity of the Recombinant LipHu6

Next, to gain more accurate data about the activity of LipHu6 with or without fluorescent protein tags, the target proteins were purified with Ni-NTA affinity (Supplementary Figure S3) and their hydrolytic activities to *p*-NP fatty acyl esters with various lengths of the acyl chain were investigated (Table 1). Among all the substrates tested, LipHu6 displayed the highest activity to *p*-NPB (C4), with a specific activity of 646,682.69 U/mmol. N-terminal-fused sfGFP_(-15)_ and C-terminal-fused mScarlet3 inhibited the activity of LipHu6 with short-chain lipid as the substrates. Interestingly, mScarlet3 displayed no obvious effect on the hydrolytic activity of LipHu6 to *p*-NPB when fused to the N-terminal of LipHu6, with a specific activity of 669,151.75 U/mmol. On the other hand, LipHu6 demonstrated relatively weaker activity as the acyl chain substrates lengthened from C4 to C18. All fusion tags promoted the hydrolytic activity of LipHu6 to long-chain *p*-NP fatty acyl esters from C12 to C18, especially LipHu6 fused with mScarlet3 tag. Their catalytic activities to *p*-NPP and *p*-NPS were approximately 1.5- and 1.7-fold higher than LipHu6, respectively.

### 3.5. The Effect of mScarlet3 on the Kinetic of LipHu6

The mScarlet3 fusion tags had no obvious effect on the optimal pH and temperature of LipHu6. However, the catalytic activity of mScarlet3-LipHu6 was higher than LipHu6-mScarlet3 (Table 2). Therefore, the kinetic parameters of these two fusion proteins were investigated. Although the catalytic turnover of both proteins was similar, the affinity of mScarlet3-LipHu6 to *p*-NPB was approximately 1.5-fold higher than LipHu6-mScarlet3, leading to an approximately 1.7-fold increase in *k_cat_/K_M_* in comparison with LipHu6-mScarlet3 (Table 2, Supplementary Figure S4). We proposed that the triad of LipHu6 (S115-H192-D116) is located at the C-terminus of LipHu6 (Supplementary Figure S5). Therefore, mScarlet3 may affect the entrance of the substrate to the active center of LipHu6 and decrease the activity of the enzyme.

## 4. Discussions

Secretion expression mitigates cellular toxicity and stress caused by foreign proteins in host cells. Additionally, it reduces downstream purification costs [25]. Consequently, this approach is widely adopted for the large-scale production of industrial enzymes. Studies on secretion expression pathways across various model organisms indicate that signal peptides are critical for the efficient translocation of target proteins from the cytoplasm to the extracellular environment [26,27]. Well-known signal peptides, such as the MF-α signal peptide in *P. pastoris* [28,29], and the LipA signal peptide in *B. subtilis* [30], have provided plenty of evidence about successful secretion expression. In *E. coli*, several natural signal sequences, including OmpA [31,32], OmpT [33] and PelB [34], facilitate the efficient translocation of heterologous polypeptides across the inner membrane when fused to their N-terminus. Meanwhile, key factors in the secretary pathways, such as SecB, DnaK-DnaJ and GroEL-GroES, were overexpressed to facilitate the secretion of the foreign genes [35]. However, there are still lacking an efficient strategy for establishing a universal platform for efficient secretion expression in *E. coli* [16,36]. Our previous reports indicated that mutants of sfGFP bearing net negative charges mediate the secretion expression of the foreign proteins [37]. Mutants of sfGFP do not function as conventional signal peptides. Firstly, they are not cleaved during translation or secretion processes. Additionally, sfGFP mutants facilitate the secretion of target proteins when fused to either the N- or C-terminus. Moreover, in most cases, the fusion protein remains retained within the cells and is released upon washing with PBS. Furthermore, Zhang et al. applied this sfGFP-mediated secretion system to Gram-positive bacteria, achieving successful secretory expression of phospholipase D with *B. subtilis* as host in 2021 [38]. Given the distinct membrane structures and components of Gram-positive and Gram-negative bacteria, identifying a universal mechanism for this secretion expression system remains challenging. To date, only certain characteristic features have been identified from successful cases. A β-barrel structure with net negative charges is essential for this system. Many fluorescent proteins in nature possess a β-barrel structure, making them potential candidates for developing secretion expression systems. One reason for selecting mScarlet3 is its monomeric nature. Most fluorescent proteins and their mutants, such as mCherry and DsRed, tend to form dimers, which may impair the activity or cause aggregation of the target protein [17]. Another reason for selecting mScarlet3 is its rapid maturation. Our previous study demonstrated that sfGFP can mediate the secretion of target proteins, whereas enhanced green fluorescent protein (EGFP) failed to achieve this objective [37]. This result implied the importance of the folding efficiency of the fluorescent protein to successful secretion.

The properties of target proteins also play a key role in successful secretion expression. LipHu6 is a small protein, about 25 kDa, with a pI of 4.9. Similarly, the RM lipase from our previous study is around 25 kDa with a pI of 4.8. More importantly, both lipases show strong hydrolytic activity toward short-chain fatty acid esters. This activity may disrupt the cell’s phospholipid bilayer, helping to release the target protein from the cytoplasm to the periplasm.

## 5. Conclusions

We present a novel fluorescent protein to mediate the *E. coli* secretion expression system in the present study. It demonstrates great potential in the industrial preparation of lipases. Moreover, it enriches our knowledge, showing that the fast-folding β-barrel structure is able to mediate the secretion expression of foreign proteins in *E. coli*.

## Figures and Tables

**Table 1:** The relative activity of LipHu6 with or without fusion tags to different substrates.

Substrate	LipHu6	sfGFP_(-15)_-LipHu6	mScarlet3-LipHu6	LipHu6-mScarlet3
*p*-NPA	58.7 ± 2.0	19.0 ± 1.9	47.3 ± 0.8	31.7 ± 3.9
*p*-NPB	100.0 ± 1.0	76.1 ± 3.0	103.4 ± 5.2	88.2 ± 2.1
*p*-NPO	29.0 ± 0.8	22.1 ± 4.0	26.9 ± 2.1	17.3 ± 1.5
*p*-NPL	13.8 ± 1.0	14.6 ± 7.2	20.8 ± 3.4	14.3 ± 4.3
*p*-NPP	29.5 ± 3.4	35.2 ± 5.8	41.7 ± 1.8	43.8 ± 4.1
*p*-NPS	28.1 ± 2.2	31.9 ± 4.3	48.7 ± 2.6	45.9 ± 1.1

Note: The enzyme activity of LipHu6 to p-NPB was defined as 100%. The relative activities of LipHu6 with or without fusion tags to different substrates were calculated as the mean and standard error of three trials.

**Table 2:** LipHu6-mScarlet3 and mScarlet3-LipHu6 kinetics for *p*-NPB.

Enzyme	*k_cat_* (s^−1^)	*K_m_* (mmol·L^−1^)	*k_ca_*_t_/*K_m_* (mmol^−1^·L·s^−1^)
mScarlet3-LipHu6	29.82 ± 3.56	0.57 ± 0.08	52.27 ± 6.28
LipHu6-mScarlet3	25.92 ± 2.31	0.85 ± 0.03	30.53 ± 2.01

Note: The experiment was performed in triplicate, and standard deviations are indicated.

## Data Availability

The original contributions presented in this study are included in the article/Supplementary Materials. Further inquiries can be directed to the corresponding author(s).

## Supplementary Materials

The following supporting information can be downloaded at: https://www.mdpi.com/article/10.3390/biom15060842/s1, Figure S1: The amino acid sequence of mScarlet3; Figure S2: Electrostatic potential and Hydrophilic distribution of sfGFP_(-15)_ and mScarlet3; Figure S3: Purification of LipHu6 with or without fluorescent protein tags; Figure S4: Kinetics of LipHu6 with or without fluorescent protein tags to p-NPB; Figure S5: The amino acid sequence of LipHub6.
