# Supplementary material for: Establishing a Novel E. coli Heterologous Secretion Expression System Mediated by mScarlet3 for the Expression of a Novel Lipolytic Enzyme
Source: Biomolecules. 2025 Jun 9;15(6):842. doi: 10.3390/biom15060842 (PMC12191403; doi:10.3390/biom15060842)
Supplement: Supplementary file 1 [file biomolecules-15-00842-s001.zip › biomolecules-3675591-supplementary.pdf]

Supplementary material

```
       10         20         30         40         50         60
MDSTEAVIKE FMRFKVHMEG SMNGHEFEIE GEGEGRPYEG TQTAKLRVTK GGPLPFSWDI

       70         80         90        100        110        120
LSPQFMYGSR AFTKHPADIP DYWKQSFPEG FKWERVMNFE DGGAVSVAQD TSLEDGTLIY

       130        140        150        160        170        180
 KVKLRGTNFP PDGPVMQKKT MGWEASTERL YPEDVVLKGD IKMALRLKDG GRYLADFKTT

       190        200        210        220        230
 YRAKKPVQMP GAFNIDRKLD ITSHNEDYTV VEQYERSVAR HSTGGSGGS
```

Supplementary Figure S1. The amino acid sequence of mScarlet3.

.

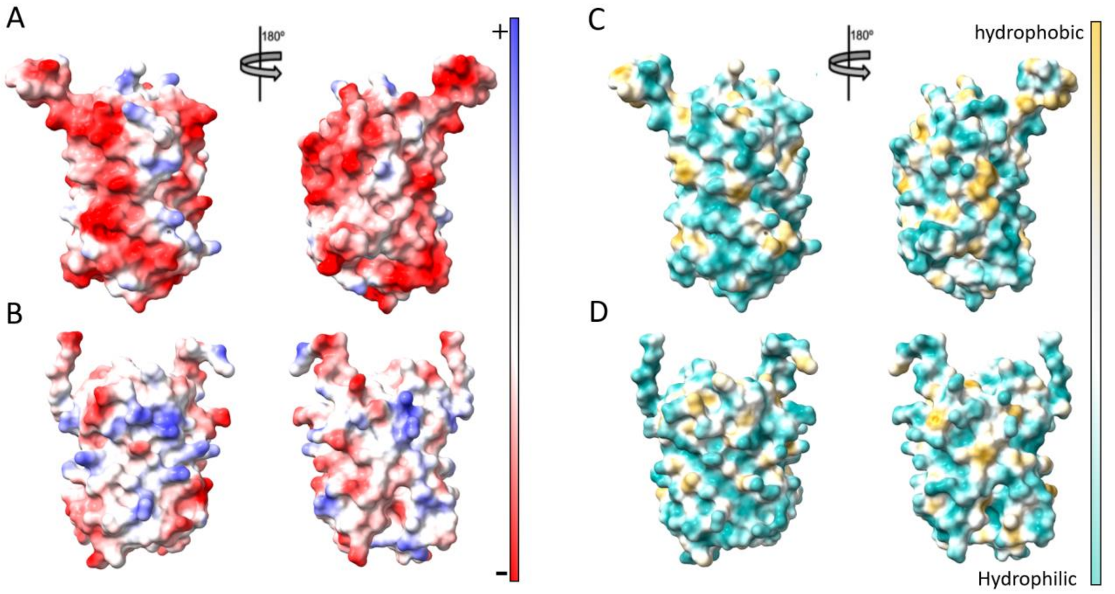

Supplementary Figure S2. Electrostatic potential and Hydrophilic distribution of sfGFP$_{(-15)}$ and mScarlet3. (A) The charge distribution on the protein surface of sfGFP$_{(-15)}$; (B) The charge distribution on the protein surface of mScarlet3; (C) Hydrophobic and hydrophilic distribution on the protein surface of sfGFP$_{(-15)}$; (D) Hydrophobic and hydrophilic distribution on the protein surface of mScarlet3.

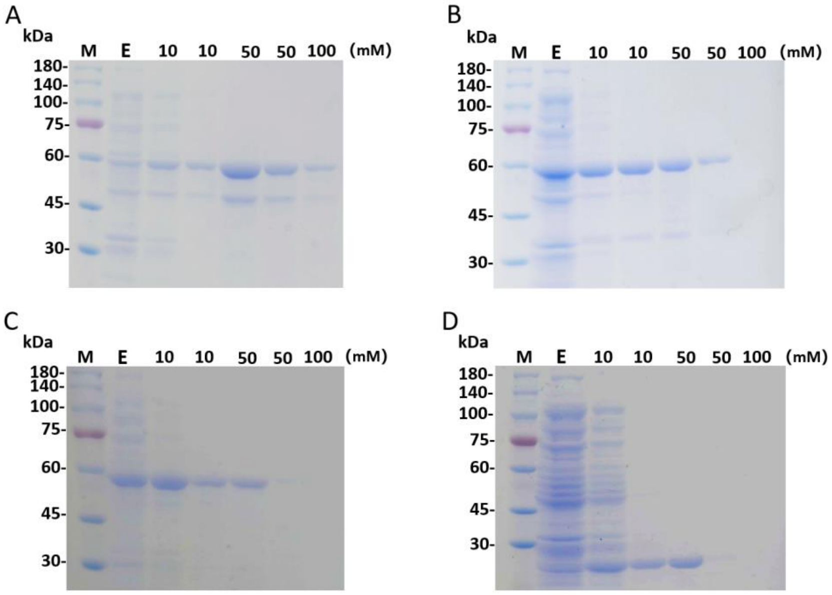

Supplementary Figure S3. Purification of LipHu6 with or without fluorescent protein tags.

(A) mScarlet3-LipHu6; (B) LipHu6-mScarlet3; (C) sfGFP$_{(-15)}$-LipHu6; (D) LipHu6. M. protein molecular weight marker (the size of each band was indicated on the left). E stands for the elute of the cell lysis, 10-100 mM are the concentrations of imidazole used for the elution of the target proteins.

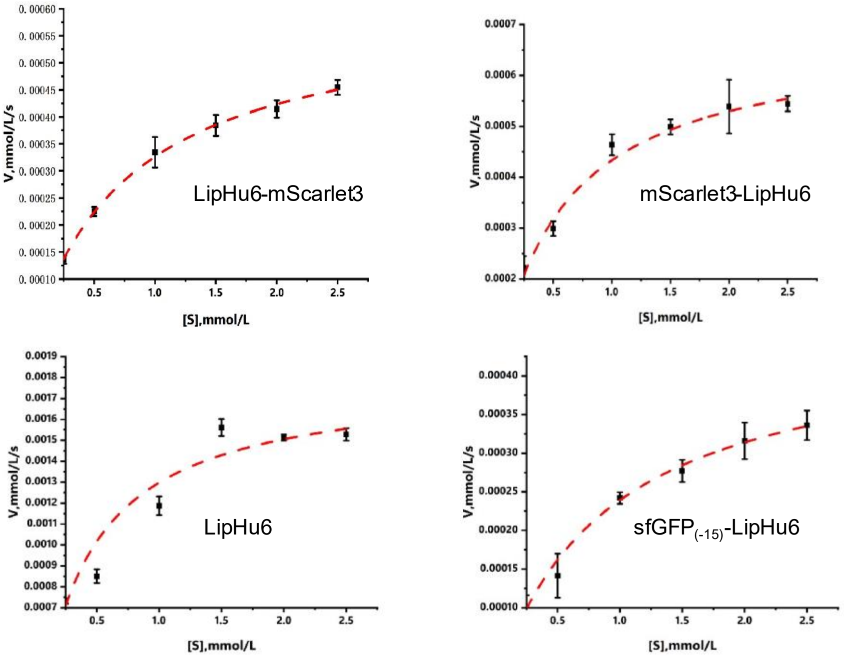

Supplementary Figure S4. Kinetics of LipHu6 with or without fluorescent protein tags to p-NPB.

```
        10         20         30         40         50         60
CEPSGVSQYD VSTGWFDGVS SARVFYPTNI NELNDVGATT LSSGMGGTKE GMYWLAEPLA

        70         80         90        100        110        120
QSGMVVVAVS ASDNMTVAGY ERSHKGGLDI LESENTDSGS PLYGKLGNRG IIGYSKGGGA

       130        140        150        160        170        180
VINVASELGD EVSTCVALAP WNPNPTYNHK AATMILTGTL DAIAPAYMGA GAYDDLPAGV

       190        200        210        220        230        240
PKLYASMVGE AHLYWNNLSN TGSETEFIVS WLKYYLEGDE AYYEVFSQGA GSGMTDYEFD

       250
PATGGGGSGG CN
```

Supplementary Figure S5. The amino acid sequence of LipHub6. The S115-H-192-D161 traid is labeled with red color and the GXSXG motif is highlighted.

.
